# Neferine mitigates angiotensin II-induced atrial fibrillation and fibrosis via upregulation of Nrf2/HO-1 and inhibition of TGF-β/p-Smad2/3 pathways

**DOI:** 10.18632/aging.205829

**Published:** 2024-05-21

**Authors:** Xiao-Xiao Jiang, Ri Zhang, Hui-Shan Wang

**Affiliations:** 1 Department of Cardiology, Institute of Cardiovascular Diseases, First Affiliated Hospital of Dalian Medical University, Dalian 116011, China; 2 State Key Laboratory of Frigid Zone Cardiovascular Disease, Department of Cardiovascular Surgery, General Hospital of Northern Command, Shenyang 110016, Liaoning, China

**Keywords:** neferine, angiotensin II, oxidative stress, atrial fibrillation, heme oxygenase-1

## Abstract

Background: Atrial fibrillation (AF) is often associated with atrial fibrosis and oxidative stress. Neferine, a bisbenzylisoquinoline alkaloid, has been reported to exert an antiarrhythmic effect. However, its impact on Angiotensin II (Ang II) infusion-induced AF and the underlying mechanism remains unclear. This study aimed to investigate whether neferine alleviates Ang II-induced AF and explore the underlying mechanisms.

Methods: Mice subjected to Ang II infusion to induce AF were concurrently treated with neferine or saline. AF incidence, myocardial cell size, fibrosis, and oxidative stress were then examined.

Results: Neferine treatment inhibited Ang II-induced AF, atrial size augmentation, and atrial fibrosis. Additionally, we observed that Ang II increased reactive oxygen species (ROS) generation, induced mitochondrial membrane potential depolarization, and reduced glutathione (GSH) and superoxide dismutase (SOD) levels, which were reversed to some extent by neferine. Mechanistically, neferine activated the Nrf2/HO-1 signaling pathway and inhibited TGF-β/p-Smad2/3 in Ang II-infused atria. Zinc Protoporphyrin (ZnPP), an HO-1 inhibitor, reduced the anti-oxidative effect of neferine to some extent and subsequently abolished the beneficial effect of neferine on Ang II-induced AF.

Conclusions: These findings provide hitherto undocumented evidence that the protective role of neferine in Ang II-induced AF is dependent on HO-1.

## INTRODUCTION

Atrial fibrillation (AF) is a serious arrhythmia disorder with an escalating incidence and morbidity globally. The progression of AF is significantly influenced by structural and electrical remodeling processes [1]. Atrial structural remodeling associated with AF induced by Angiotensin II (Ang II) is characterized by increased atrial dilation, fibrosis, oxidative stress, and inflammatory responses [2]. Prior research has emphasized the crucial role of oxidative stress in regulating structural remodeling in AF [3]. Hence, there is an urgent need to identify novel drugs that can inhibit oxidative stress for the effective treatment of AF.

Effective therapeutic interventions for AF involve genetic or pharmacological inhibition of oxidative enzymes or strategies to upregulate antioxidant enzymes, thereby reducing reactive oxygen species (ROS) in atrial myocytes [4]. Nuclear factor erythroid 2-related factor 2 (Nrf2) serves as a vital regulator, protecting against oxidative injury by regulating redox signaling. Current evidence suggests that inhibition of Nrf2 diminishes antioxidant signaling, promoting atrial remodeling and fibrillation [5]. Heme oxygenase-1 (HO-1), an antioxidant enzyme, facilitates the breakdown of heme into iron, carbon monoxide, and biliverdin, which is further converted into bilirubin [6]. HO-1's effects include the reduction of ROS generation and alleviating oxidative stress-related conditions such as AF [4, 7, 8]. Recent studies have indicated that drugs activating the Nrf2/HO-1 signaling pathway suppress cellular remodeling induced by atrial tachypacing and may protect against AF [9]. Thus, upregulating HO-1 expression to prevent oxidative stress represents an effective therapeutic strategy for treating Ang II-induced AF.

Neferine, a bisbenzylisoquinoline alkaloid isolated from the seeds of *Nelumbo nucifera* Gaertn, has exhibited various pharmacological actions, including anti-arrhythmic, antiplatelet, anti-thrombotic, anti-hypertensive, and vascular smooth muscle relaxation effects [10]. Recent studies have highlighted neferine's antioxidant and anti-inflammatory effects, primarily attributed to its inhibitory impact on the MAPK and NF-κB/IκBα pathways [11]. Notably, neferine has been shown to increase the content of antioxidant enzymes, including HO-1, glutathione peroxidase (GSH-PX), superoxide dismutase (SOD), and catalase (CAT), in the liver and smooth muscle cells [11, 12]. However, whether neferine plays a protective role in Ang II-induced AF and the underlying molecular mechanism remains unclear.

This study aims to investigate the effects and mechanisms of neferine on Ang II-induced AF, oxidative stress, and atrial structural remodeling.

## MATERIALS AND METHODS

### Antibodies and reagents

Nrf2, HO-1, TGF-β, Smad-2, Smad-3, Collagen I, Collagen III, and GAPDH antibodies were purchased from Proteintech (Wuhan, China). The p-Smad2/3 antibody was purchased from Cell Signaling Technology (Danvers, MA, USA). Angiotensin II and ROS fluorescent probe DHE were obtained from Aladdin (Shanghai, China), and the neferine and zinc protoporphyrin (ZnPP, HO-1 inhibitor) were obtained from MedChemExpress (Monmouth Junction, NJ, USA). The mito-SOX Red was obtained from Invitrogen (Thermo Fisher Scientific, USA, cat#: M36008). The superoxide dismutase and malondialdehyde (MDA) assay kits were obtained from Solarbio (Beijing, China). The glutathione (GSH) assay kit and JC1 mitochondrial membrane potential assay kit were obtained from Beyotime Biotechnology (Shanghai, China). The atrial natriuretic peptide (ANP) and brain natriuretic peptide (BNP) ELISA kits were obtained from Elabscience Biotechnology (Wuhan, China).

### Animals and ethics statement

The C57BL/6 mice were obtained from Liaoning Changsheng BioTechnology Company (Benxi, Liaoning, China). The mice were handled under the operating rules of the University of Dalian Medical University Animal Welfare Regulations. All mice were housed in a controlled environment with a temperature range of 22° C–24° C and a 12-hour light/dark cycle. All experimental procedures involving mice received approval from the ethics committee of Dalian Medical University.

### Ang II infusion and neferine administration

Mice were subjected to a 4-week infusion of either saline or Ang II (2000 ng/kg/min) using osmotic mini-pumps (Alzet MODEL 1004; DURECT). Neferine preparation involved dissolving neferine powder in 10% DMSO, followed by mixing with 40% PEG300, 5% Tween-80, and 45%. Mice treated with saline or Ang II received neferine (60 mg/kg/day) or the control vehicle via gavage for the same 4-week period. The HO-1 inhibitor, ZnPP, was dissolved in 0.2 M NaOH, pH adjusted to 7.4 with hydrochloric acid, and then diluted with saline to a concentration of 1 mg/ml. For ZnPP treatment, mice were pretreated with ZnPP (10 mg/kg body weight) or the vehicle 1 hour before Ang II and neferine administration. After the 4-week drug treatments, atrial pacing was employed to induce atrial fibrillation, and subsequently, atrial tissues were collected for molecular biological analysis.

### Echocardiography

Following drug treatment, mice were subjected to anesthesia induction with 2% isoflurane mixed with O_2_ via inhalation. Atrial structural changes in mice were assessed using trans-thoracic two-dimensional M-mode echocardiography conducted with the Vevo 770 imaging system (VisualSonics, Toronto, Canada). To maintain a heart rate between 500-600 beats per minute, mice were positioned on a heating pad after shaving. A two-dimensional guided M-mode trace, obtained from the parasternal short axis intersecting the papillary muscle level using a 30 MHz transducer, was recorded at a depth of 2 cm and a sweep speed of 100 mm/s. The left atrial diameter was measured in the parasternal long-axis view, spanning from the anterior to posterior walls. Image analysis was performed using VevoLab software.

### AF induction

Mice were anesthetized with tribromoethanol (180 mg/kg, Sigma-Aldrich, St. Louis, MO, USA), and a Millar 1.1F octapolar EP catheter (Scisense) was then inserted into the right atrium and ventricle. Following established procedures (as previously described), an automated stimulator was utilized to induce AF at voltages of 3.5, 5, and 8 V [13]. The AF electrograms were recorded using a computer-based data acquisition system (GY6328B; HeNan HuaNan Medical Science and Technology, Co., Ltd., China). Following 5-second bursts, mice exhibited rapid and fragmented atrial electrograms accompanied by abnormal atrioventricular (AV)-nodal conduction and ventricular rhythm lasting for more than 1 second, which was identified as the occurrence of AF. Then mice were euthanized and followed by perfusion with 10 ml of PBS and removal of the atrium into either 4% paraformaldehyde or into liquid nitrogen for freezing and long-term storage.

### Cell culture and drug treatment

HL-1 cells were cultured in Claycomb medium supplemented with 10% fetal bovine serum, 4 mM L-glutamine, and 100 μM norepinephrine at 5% CO_2_ and 37° C. Upon reaching 90% confluency, the cells underwent a 4-hour period of serum starvation in a culture medium without fetal bovine serum (FBS) before exposure to neferine at concentrations of 0, 2, 5, and 10 μM for a duration of 24 hours. For Angiotensin II (Ang II) treatment, cells were incubated with 100 nM Ang II in the presence or absence of 10 μM neferine for 24 hours, after which they were subjected to a commercial assay kit.

### Measurement of plasma ANP and BNP levels

After euthanizing the mice, blood samples were promptly collected into microcentrifuge tubes containing aprotinin (750 units/mL) and ethylenediaminetetraacetic acid disodium salt (1.8 mg/mL). Following centrifugation, the plasma obtained was stored at −80° C until assay. Plasma levels of ANP and BNP were quantified using an ELISA kit (Elabscience Biotechnology, Wuhan, China) in accordance with the manufacturer's instructions.

### DCF-DA staining

Following Ang II and neferine treatment, HL-1 cells underwent three washes with PBS. Subsequently, they were stained with a 1× DCF-DA working solution at 37° C for 30 minutes. After another three washes with PBS, images were captured using a fluorescence microscope (Olympus BX51, Tokyo, Japan). The green fluorescence intensity, indicative of ROS generation, was quantified using Image-J software.

### Assessment of ROS and mitochondrial membrane potential (MMP)

To detect mitochondrial membrane potential, the dye JC-1 was employed. After drug treatment, HL-1 cells were washed with PBS and incubated with DMEM medium containing JC-1 staining reagent at 37° C for 20 minutes. Subsequently, the cells were washed with PBS to remove any excess dye. Mitochondrial depolarization was indicated by an increase in the green/red fluorescence intensity ratio. The mitochondrial membrane potential was then detected using a fluorescence microscope (Olympus BX51, Tokyo, Japan), and the fluorescence intensity was analyzed using Image J software.

### GSH content assay

The GSH content was assessed using a total GSH assay kit (Beyotime Biotech) following a previously described protocol [14]. Tissue samples (100 μg) were lysed in the protein removal solution S provided in the kit. Subsequently, the samples were centrifuged at 12,000 g for 15 minutes at 4° C to collect the supernatant. After mixing the samples with Ellman’s reagent (DTNB), NADPH, and GSH reductase enzyme, the GSH concentration was measured at 412 nm using a microplate reader (Tecan Infinite Pro, Switzerland).

### SOD and MDA measurement

The atrial tissue was homogenized using ultrasonication in an appropriate buffer and then centrifuged at 1,500 rpm and 4° C for 5 minutes. Subsequently, the supernatant was removed. The levels of superoxide dismutase and malondialdehyde in the atrial tissue of each group were determined using a microplate reader following the instructions provided in their respective assay kits.

### Masson’s trichrome and sirius red staining

The atrial tissues were fixed in formalin for 48 hours at room temperature, followed by dehydration in various concentrations of ethanol and xylene, and ultimately embedded in paraffin. Upon obtaining 5-mm serial sections, the sections were stained with Masson’s trichrome (Solarbio, Beijing, China) according to the provided instructions. For Sirius red staining, sections were treated with Sirius red solution for 1 hour at room temperature. Images of each sample were captured using an Olympus BX51 microscope (Tokyo, Japan), and the atrial fibrosis area was quantified using Image J software.

### DHE and wheat germ agglutinin (WGA) staining

To evaluate intracellular ROS generation, fresh atrial tissue was incubated with a 50 μM dihydroethidium (DHE) working solution at 37° C for 30 minutes in a dark box. After incubation, the tissue sections were washed with warm PBS buffer three times for 5 minutes each, and relevant images were immediately captured using a fluorescence microscope (Olympus BX51, Tokyo, Japan). The red fluorescence observed represented ROS generation in the atrium. For the assessment of cardiomyocyte size, heart sections were stained with 50 μg/ml TRITC-labeled WGA in PBS buffer at 37° C for 1 hour. After staining, the sections were washed three times with warm PBS. Images from each sample were photographed using an Olympus BX51 microscope (Tokyo, Japan), and the myocardial size was calculated using Image J software.

### Quantitative real-time PCR analysis

Atrial tissues were homogenized in TRIzol (Invitrogen, Carlsbad, CA, USA), and total RNA was extracted following the manufacturer’s protocol. After assessing the RNA concentration, cDNA was synthesized using a Reverse Transcriptase Kit (Takara) with a Thermal Cycler (Thermo Fisher Scientific, Waltham, MA, USA). The primer sequences for all genes (synthesized by Shanghai Sangon Biotech) were as follows: ANP (forward: 5'-TACAGTGCGGTGTCCAACACAG-3′; reverse: 5′-TGCTTCCTCAGTCTGCTCACTC-3′), BNP (forward: 5′-TCCTAGCCAGTCTCCAGAGCAA-3′; reverse: 5′-GGTCCTTCAAGAGCTGTCTCTG-3′), Collagen I (forward: 5′-GAGTACTGGATCGACCCTAACCA-3′; reverse: 5′-GACGGCTGAGTAGGGAACACA-3′), Collagen III (forward: 5′-TCCCCTGGAATCTGT GAATC-3′; reverse: 5′-TGAGTCGAATTGGGGAGAAT-3′), and GAPDH (forward: 5’-GGTTGTCTCCTGCGACTTCA-3’and reverse: 5’-GGTGGTCCAGGG TTTCTTACTC-3’). GAPDH gene was used as the loading control, all target genes expression were normalized to GAPDH. The values were analyzed by ^2-ΔΔ^CT method, and finally the data was shown as the fold change.

### Western blotting

The atrial tissue was lysed in ice-cold RIPA buffer (containing 1% PMSF), followed by centrifugation at 4° C for total protein extraction. After mixing 40 μg of total protein samples with loading buffer and boiling at 100° C for 5 minutes, the proteins were subjected to SDS-PAGE gel for electrophoresis to isolate proteins. The gels were then transferred onto PVDF membranes at 300 mA for 90 minutes. Subsequently, 5% skim milk in TBS-Tween buffer (0.2% Tween 20; pH 7.5) was used to block the membranes, and the membranes were incubated with primary antibodies, including Nrf2, HO-1, TGF-β, p-Smad2/3, Smad-2, Smad-3, and GAPDH overnight at 4° C. On the following day, the membranes were washed with TBST buffer and subsequently incubated with the relevant secondary antibodies for 1 hour at room temperature. The protein bands were visualized using a Protein Simple Imager (Protein Simple, San Jose, CA, USA), and the band intensity was quantified using Image J software.

### Statistical analysis

The data were analyzed using GraphPad Prism software version 8.0. Comparisons between multiple groups were assessed through one-way multivariate ANOVA, followed by a Tukey post hoc test or a Student t-test where applicable. A p-value ≤ 0.05 was statistically significant.

### Data availability

Data will be made available on request.

## RESULTS

### Neferine inhibited Ang II-induced AF and atrial dilation in mice

**Figure 1:**
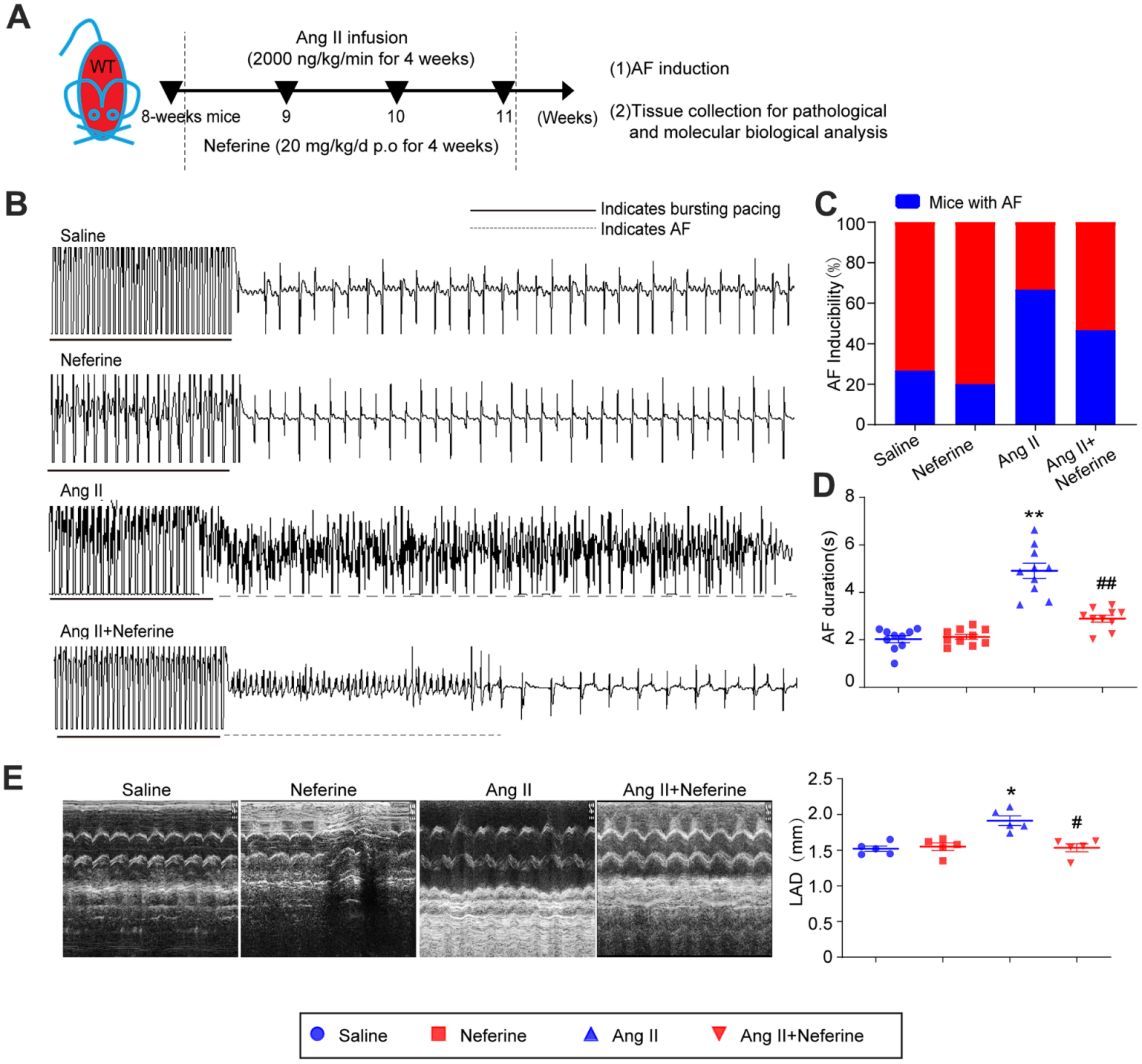
**Neferine inhibited Ang II-induced AF and atrial dilation in mice.** (**A**) 8-week-old mice were infused with saline or Ang II (2000ng/kg/min) and received vehicle or neferine (20 mg/kg) each day for 4 weeks (*n* = 10 samples per group). (**B**, **C**) Electrocardiogram of the occurrence of AF stimulated by burst pacing and the incidence of AF in each treatment group (n = 10). (**D**) Average AF duration induced by burst pacing (n = 10). (**E**) Echocardiography showed representative images of two-dimensional M-mode for the measurement of left atrium dimension (LAD, mm). Data are presented as mean ± SEM, and n represents the number of samples per group. *P < 0.05 for the difference compared with the Saline group; ^#^*P* < 0.05 for the difference compared with the Ang II group.

To investigate the impact of neferine on Ang II-induced AF, 8-week-old mice were subjected to either saline or Ang II (2000 ng/kg/min) infusion, concomitantly receiving equivalent vehicle or neferine (20 mg/kg) daily for a duration of 4 weeks (Figure 1A). The study revealed that Ang II infusion over 4 weeks significantly increased AF incidence and duration following pacing stimulation, while treatment with neferine mitigated this effect (Figure 1B–1D). Structural changes were assessed through echocardiography to examine alterations in atrial morphology. The results demonstrated that Ang II infusion notably induced atrial dilation, a phenomenon partially alleviated by neferine treatment (Figure 1E). These findings suggest that neferine possesses the ability to attenuate both Ang II-induced AF and structural remodeling.

### Neferine inhibited Ang II-induced atrial cell hypertrophy and fibrosis

**Figure 2:**
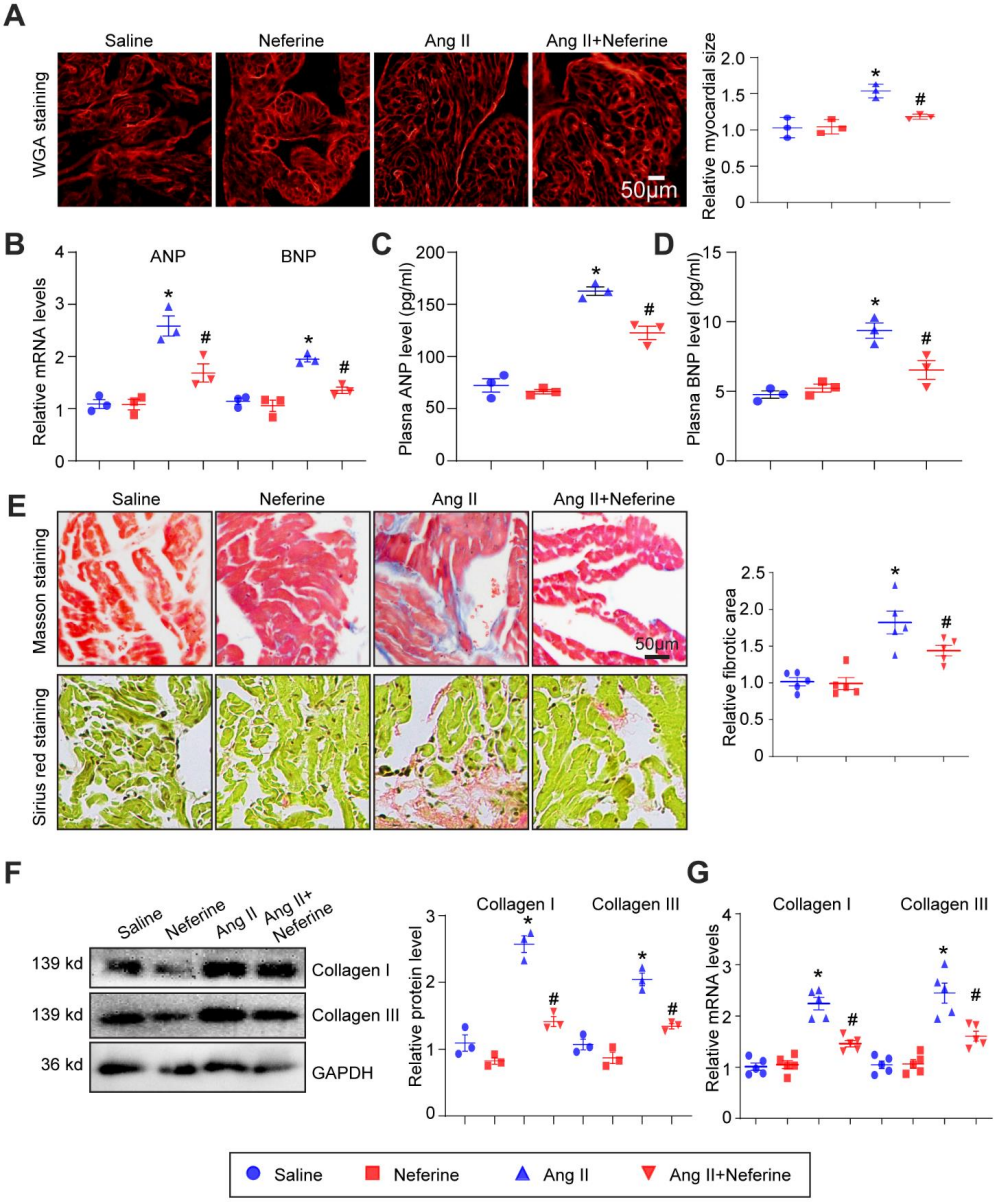
**Neferine inhibited Ang II-induced atrial cell size augmentation and atrial fibrosis.** (**A**) Representative images of WGA staining illustrating atrial cell size in each group (n = 3, on the right). Scale bar: 50 μm. (**B**) Measurement of ANP and BNP mRNA levels by qPCR (n = 3). (**C**, **D**) Plasma ANP and BNP levels assessed using an Elisa kit. (**E**) Representative images of Masson's trichrome and Sirius Red-stained atrial sections depicting the degree of fibrosis; the fibrotic area in each group was quantified (n = 5, on the right). Scale bar: 50 μm. (**F**) Western blot analysis of collagen I and III protein expression in the atria, with corresponding statistical results on the right (*n* = 3). (**G**) Measurement of collagen I and III mRNA levels by qPCR (n = 5, on the right). Data are presented as mean ± SEM, and n represents the number of samples. *P < 0.05 for the difference compared with the Saline group; ^#^*P* < 0.05 for the difference compared with the Ang II group.

As we previously reported, atrial cell size augmentation, fibrosis, and oxidative stress typically occur in Ang II-infused atrium during AF [15]. Consequently, we sought to investigate whether neferine could offer protection against Ang II-induced atrial cell hypertrophy and fibrosis, employing WGA, Masson's trichrome, and Sirius red staining techniques. Neferine administration notably hindered the Ang II-induced atrial cell hypertrophy (Figure 2A), and the upregulation of cardiac hypertrophic biomarkers ANP and BNP mRNA, as well as the increased plasma ANP and BNP concentrations observed in Ang II-infused mice, were also suppressed following neferine administration for a duration of 4 weeks (Figure 2B–2D). Furthermore, we observed that Ang II-induced atrial fibrosis was partially alleviated by neferine, as evidenced by Masson's trichrome and Sirius red staining of atrial sections (Figure 2E). Subsequently, fibrotic biomarkers such as Collagen I and Collagen III protein and mRNA levels were assayed in Ang II-infused atria. Similarly, Ang II infusion heightened Collagen I and Collagen III protein and mRNA levels, all of which were significantly reduced by neferine treatment (Figure 2F, 2G).

### Neferine inhibited Ang II-induced oxidative stress

**Figure 3:**
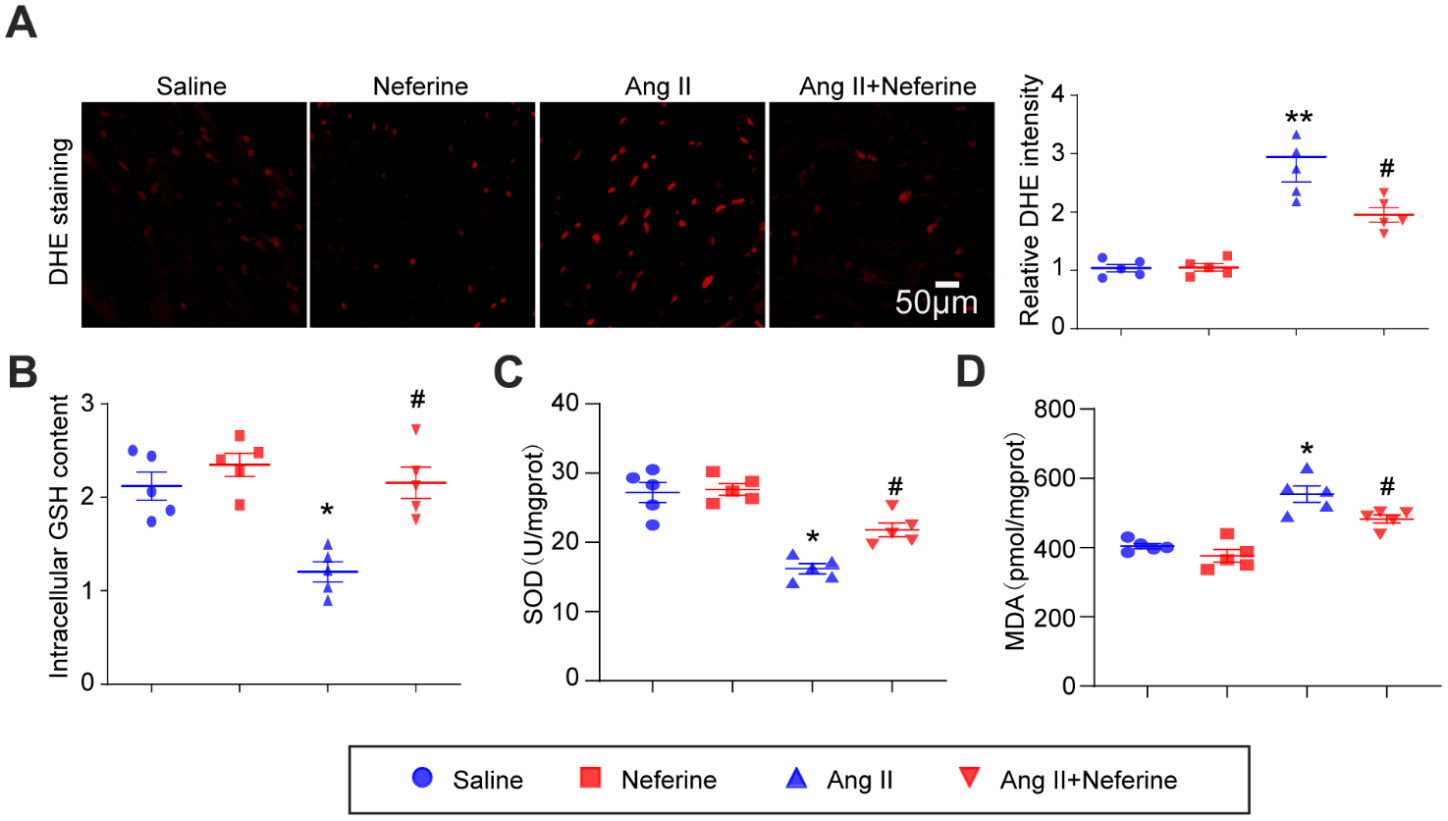
**Neferine inhibited Ang II-induced atrial oxidative stress.** (**A**) Determination of ROS generation in the atrium through DHE staining; the red fluorescence intensity was quantified and shown on the right (n = 5). (**B**) Measurement of relative atrial GSH levels (*n* = 5). (**C**) Assessment of relative atrial SOD levels (*n* = 5). (**D**) Evaluation of relative atrial MDA levels (*n* = 5). Data are presented as mean ± SEM, and n represents the number of samples. *P < 0.05 for the difference compared with the Saline group; ^#^*P* < 0.05 for the difference compared with the Ang II group.

We assessed whether neferine could mitigate Ang II-induced oxidative stress in the mice atrium. DHE staining revealed that Ang II infusion led to an increase in ROS generation, indicated by elevated red fluorescence intensity. Notably, neferine administration significantly inhibited this effect (Figure 3A). Consistent with these findings, Ang II infusion resulted in a reduction in GSH and SOD content, along with an increase in MDA content in atrial tissues. Neferine treatment markedly reversed these effects, restoring GSH and SOD levels while reducing MDA content (Figure 3B–3D). Given that GSH and SOD function to eliminate ROS, the antioxidant effect of neferine may be attributed to its ability to enhance GSH and SOD content.

### Neferine inhibited Ang II-induced ROS generation and mitochondrial membrane potential depolarization in HL-1 cells

**Figure 4:**
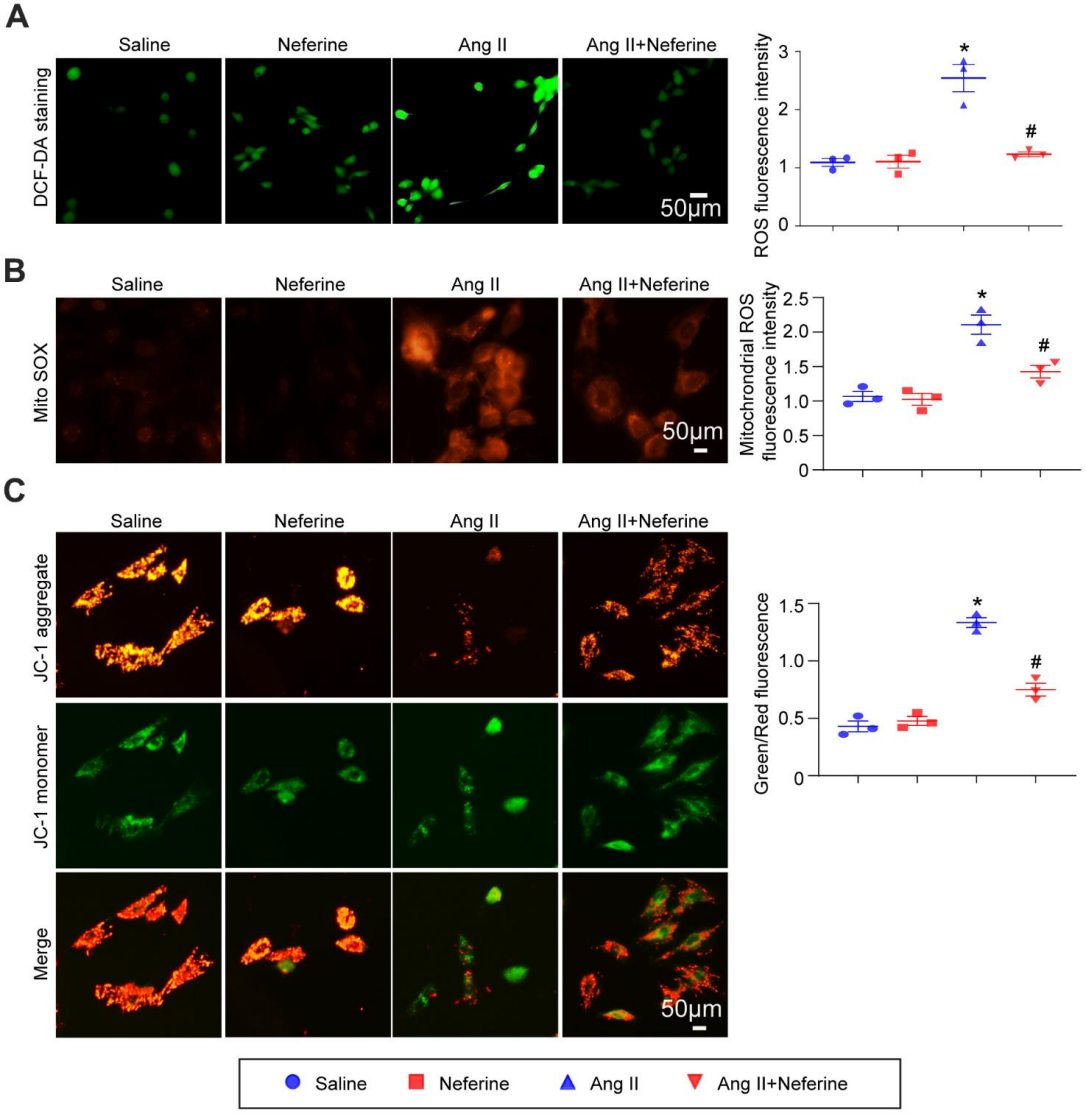
**Neferine inhibited Ang II-induced ROS generation and mitochondrial membrane potential depolarization.** (**A**) HL-1 cells were treated with 100 nM Ang II and 10 μM neferine for 24 hours; ROS generation was determined by DCF-DA staining, and the statistical results are shown on the right (*n* = 3). (**B**) Mitochondrial ROS generation was assessed through MitoSOX staining, with the corresponding statistical results on the right (n = 3). (**C**) Mitochondrial membrane potential was determined by JC-1 staining, and the statistical results are shown on the right (n = 3). *P < 0.05 for the difference compared with the Saline group; ^#^*P* < 0.05 for the difference compared with the Ang II group.

The antioxidant capacity of neferine was assessed using DCF-DA and MitoSOX Red staining in HL-1 cells. DCF-DA staining, serving as a probe for ROS generation, was applied to HL-1 cells, and the resulting fluorescence intensity was measured. Our findings indicated that Ang II treatment significantly increased ROS generation, a phenomenon attenuated by neferine treatment (Figure 4A). Subsequently, mitochondrial oxidant levels were determined by MitoSOX Red staining. The results demonstrated that neferine treatment significantly reduced mitochondrial oxidant generation compared to cells treated with Ang II alone (Figure 4B). Additionally, to assess the impact of neferine on Ang II-induced alterations in mitochondrial membrane potential, JC-1 staining was performed. Cells treated with Ang II exhibited less green fluorescence and more red fluorescence, indicative of mitochondrial depolarization. Neferine treatment significantly inhibited Ang II-induced mitochondrial depolarization, as reflected by the preservation of mitochondrial red fluorescence compared to cells treated with Ang II alone (Figure 4C).

### Neferine activated Nrf2/HO-1 signaling pathway and inhibited TGF-β/p-Smad2/3

**Figure 5:**
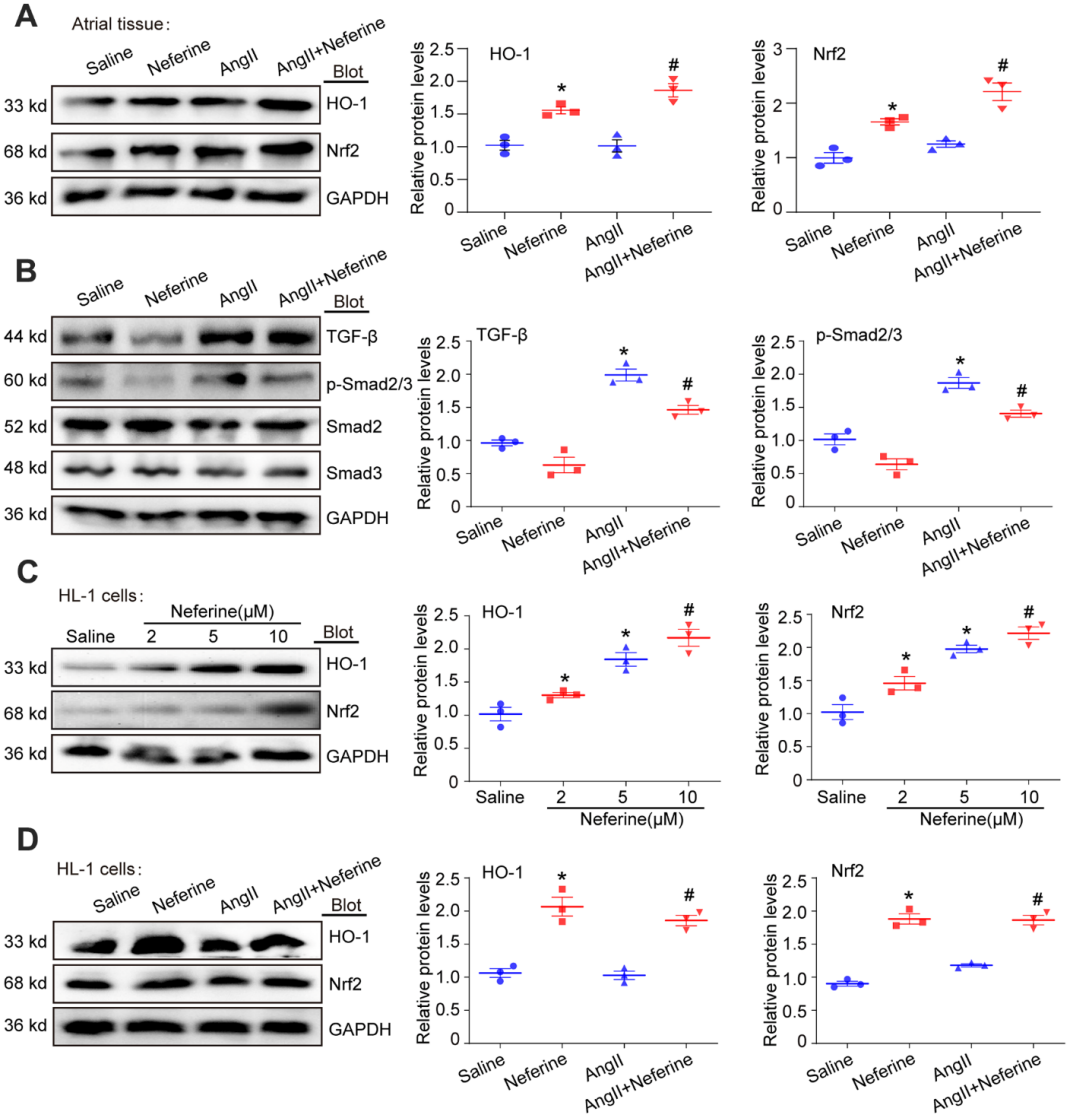
**Neferine activated Nrf2/HO-1 and inhibited TGF-β/p-Smad2/3 signaling pathway.** (**A**) Western blotting was performed to assess the expression of Nrf2 and HO-1 in the atrium, and the statistical results are shown on the right (*n* = 3). (**B**) Protein expression of TGF-β, p-Smad2/3, Smad2, and Smad3 in the atria was determined by western blot, with the corresponding statistical results on the right (*n* = 3). (**C**) HL-1 cells were treated with 2, 5, and 10 μM neferine for 24 hours, and Nrf2 and HO-1 protein expression were assessed by western blot, with the statistical results shown on the right (*n* = 3). (**D**) HL-1 cells were treated with 100 nM Ang II and 10 μM neferine for 24 hours, and Nrf2 and HO-1 protein expression were determined by western blot, with the statistical results shown on the right (*n* = 3). GAPDH served as an internal control. Data are presented as mean ± SEM, and n represents the number of samples. *P < 0.05 for the difference compared with the Saline group; ^#^*P* < 0.05 for the difference compared with the Ang II group.

Increased expression of HO-1 has been shown to reduce ROS generation, thereby inhibiting [8]. Previous reports also suggested that neferine inhibits vascular smooth muscle cell proliferation induced by Ang II through the activation of heme oxygenase-1 [12]. Hence, our investigation aimed to determine whether neferine upregulates HO-1 expression in Ang II-infused atria, subsequently inhibiting oxidative stress and AF. As expected, Western blot results revealed that Ang II did not affect Nrf2 and HO-1 expression. However, neferine administration significantly increased Nrf2 and HO-1 protein expression in the presence or absence of Ang II (Figure 5A). Cardiac fibrosis mediated by TGF-β/p-Smad2/3 activation is associated with Ang II-infused hearts. Further examination of the effects of neferine on TGF-β/p-Smad2/3 revealed a significant increase in TGF-β and p-Smad2/3 protein expression in Ang II-infused atria compared to the Saline group. Notably, these effects mediated by Ang II were partially attenuated after neferine treatment (Figure 5B). Subsequently, HL-1 cells were treated with different concentrations of neferine, revealing a dose-dependent activation of the Nrf2/HO-1 signaling pathway (Figure 5C). Consistent with *in vivo* data, neferine treatment significantly increased Nrf2 and HO-1 protein expression in the presence or absence of Ang II in HL-1 cells (Figure 5D).

### Inhibition of HO-1 by ZnPP partially abolished the protective role of neferine in Ang II-induced AF, atrial cell hypertrophy, and fibrosis

**Figure 6:**
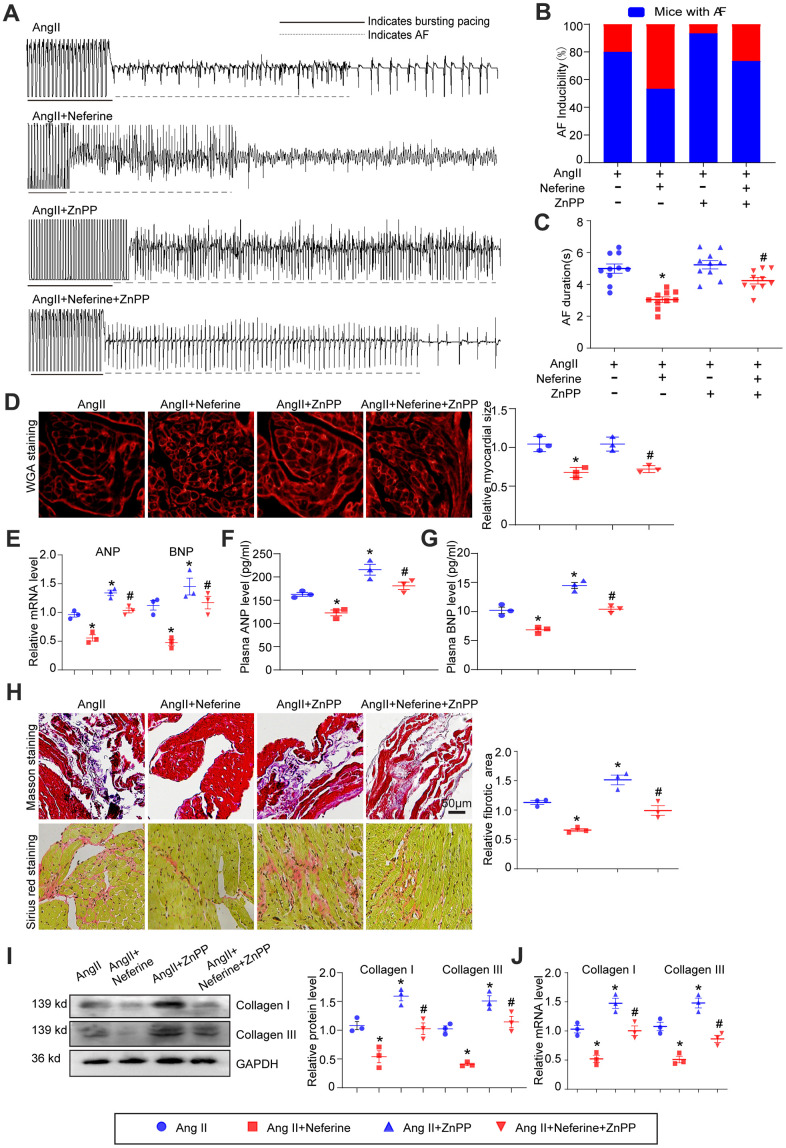
**Inhibition of HO-1 partially abolished the protective role of neferine in Ang II-induced AF, atrial cell size augmentation, and fibrosis.** (**A**) AF records in each group. (**B**, **C**) Average AF incidence and duration induced by burst pacing (n = 10). (**D**) Representative images of WGA staining of atrial sections illustrating atrial cell size, with the cell area in each group quantified (n = 3, on the right). Scale bar: 50 μm. (**E**) Measurement of ANP and BNP mRNA levels by qPCR (n = 3, on the right). (**F**, **G**) Plasma ANP and BNP levels assessed by Elisa kit (n = 3). (**H**) Representative images of Masson's trichrome and Sirius Red-stained atrial sections indicating the degree of fibrosis, with the fibrotic area in each group quantified (n = 5, on the right). Scale bar: 50 μm. (**I**) Western blot analysis of collagen I and III protein expression in the atria, with the corresponding statistical results on the right (*n* = 3). (**J**) Measurement of collagen I and III mRNA levels by qPCR (n = 3). Data are presented as mean ± SEM, and n represents the number of samples. Data are presented as mean ± SEM, and n represents the number of samples. *P < 0.05 for the difference compared with the Ang II group; ^#^*P* < 0.05 for the difference compared with the Ang II + neferine group.

To investigate whether the inhibitory effect of neferine on Ang II-induced AF is attributed to its ability to upregulate HO-1 expression, mice were pretreated with the HO-1 inhibitor ZnPP before being subjected to Ang II and neferine. Interestingly, administration of the HO-1 inhibitor ZnPP partially abolished the protective effect of neferine on Ang II-induced AF (Figure 6A). Neferine's reduction of Ang II-induced AF incidence and duration was counteracted to a certain extent by ZnPP (Figure 6B, 6C). Subsequently, the impact of ZnPP on Ang II-induced atrial cell hypertrophy and atrial fibrosis was determined. It was observed that the increase in atrial cell size induced by Ang II, initially inhibited by neferine, was markedly reversed by ZnPP (Figure 6D). Consistent with this, the mRNA expression of ANP and BNP in the atrium, as well as protein concentrations in plasma, exhibited similar changes (Figure 6E–6G). Masson's trichrome and Sirius red staining results demonstrated that neferine inhibited Ang II-induced atrial fibrosis, an effect that was markedly reversed by ZnPP (Figure 6H). Correspondingly, mice subjected to Ang II and neferine co-treatment exhibited lower protein and mRNA expression of Collagen I and Collagen III compared to Ang II infusion alone (Figure 6I, 6J). These effects were partially abolished by the presence of the HO-1 inhibitor ZnPP, indicating an essential role of HO-1 in the protective effects of neferine (Figure 6I, 6J).

### Inhibition of HO-1 by ZnPP partially abolished the protective role of neferine in Ang II-induced oxidative stress

**Figure 7:**
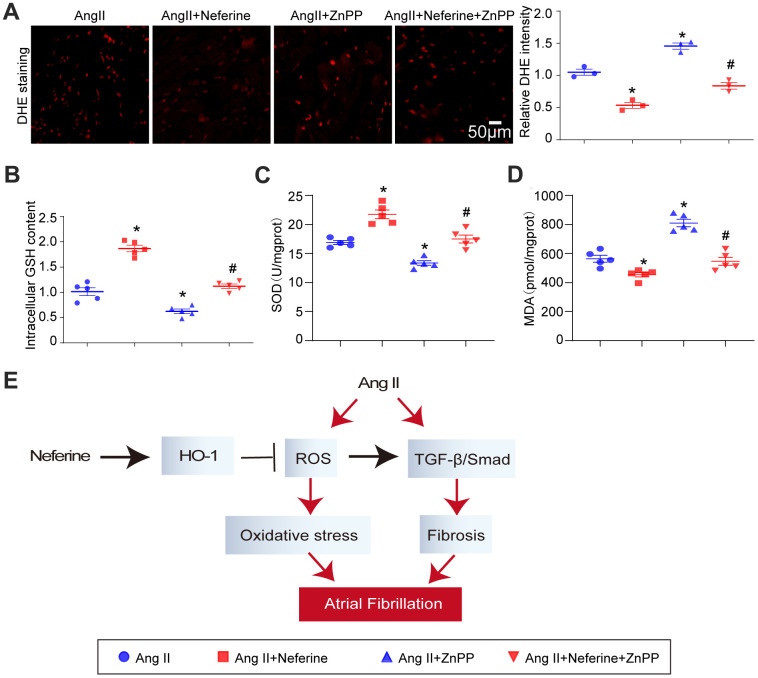
**Inhibition of HO-1 partially abolished the protective role of neferine in Ang II-induced oxidative stress.** (**A**) The ROS generation in the atrium was determined by DHE staining; the red fluorescence intensity was quantified and shown on the right (n = 3). (**B**) Relative atrial GSH level (*n* = 5). (**C**) Relative atrial SOD level (*n* = 5). (**D**) Relative atrial MDA level (*n* = 5). (**E**) The schematic diagram illustrates the mechanism of neferine in protecting AF and atrial fibrosis. Data are presented as mean ± SEM, and n represents the number of samples. *P < 0.05 for the difference compared with the Ang II group; ^#^*P* < 0.05 for the difference compared with the Ang II + neferine group.

Subsequent to the above experiments, atrial tissue was stained with DHE to evaluate ROS generation. Results indicated that neferine inhibited Ang II-induced ROS generation, and this effect was partially abolished by ZnPP treatment (Figure 7A). Furthermore, neferine's reduction in malondialdehyde content and its increase in GSH content and SOD activity in Ang II-infused atria were partially reversed by ZnPP (Figure 7B–7D). These findings collectively suggest that the protective effects of neferine are mediated, at least in part, by the Nrf2/HO-1 signaling pathway.

## DISCUSSION

Atrial fibrillation is a prevalent and severe arrhythmia disease worldwide, often leading to recurrent episodes and, in some cases, permanent AF that can induce cardiac insufficiency [16]. This study aimed to explore new drugs for preventing and treating AF. Neferine was identified as a compound that activates the Nrf2/HO-1 signaling pathway while inhibiting the TGF-β/p-Smad2/3 pathway. This dual action correspondingly inhibited Ang II-induced ROS generation and atrial fibrosis, ultimately alleviating AF induced by Ang II (Figure 7E).

Oxidative stress is increasingly recognized as a key mechanism in the pathogenesis of AF [3]. In the context of AF, tachycardia-induced elevation of ROS in ventricles leads to apoptosis [17], and oxidative stress and mitochondrial dysfunction have been detected in atrial tissue of AF patients [18]. Gene expression profiling revealed a reduction in the expression of anti-oxidative genes and an upregulation of ROS-producing genes in the atrial tissue of patients with AF [19]. Nrf2, identified as a central regulator of antioxidant genes [20], demonstrated its upstream role in HO-1 expression, as knockdown of nrf2 inhibited HO-1 expression [9]. HO-1, a crucial protein known for its protective role against oxidative stress in various cardiovascular diseases [21], played a significant role in alleviating AF and atrial fibrosis. Notably, HO-1 knockout mice exhibited greater susceptibility to AF following burst atrial pacing compared to WT mice [8, 9]. In the context of treating AF in rats, bone marrow mesenchymal stem cells overexpressing Nrf2-derived exosomes emerged as a promising approach, primarily due to their ability to reduce cardiac fibrosis [22]. Despite this progress, the specific effects of HO-1 on Ang II-induced AF pathogenesis and therapeutic potential remain unclear. Our current study addressed this gap by demonstrating that in the Ang II-induced AF model, Ang II significantly increased ROS generation while decreasing GSH and SOD content in the infused atrium. Neferine, however, countered oxidative stress by augmenting endogenous antioxidants such as GSH and SOD and concurrently reducing ROS generation. The blockade of HO-1 by ZnPP nullified these effects, indicating that HO-1 was a crucial mediator for neferine's inhibitory impact on Ang II-induced atrial remodeling. Consequently, the induction of HO-1 emerges as the essential mechanism through which neferine suppresses oxidative damage and prevents atrial structural remodeling.

Prior studies have highlighted neferine's inhibitory impact on ventricular tachyarrhythmias, potentially attributed to its ability to inhibit Na^+^, K+, and Ca^2+^ currents in the myocardium [23]. In addition, neferine has demonstrated efficacy in mitigating fibrosis in various organs, including the lung and liver [23]. Neferine treatment has shown a notable inhibitory effect on cardiac fibrosis induced by high glucose and doxorubicin [24, 25]. However, its influence on Ang II-induced atrial fibrillation (AF) and atrial fibrosis has not been explored. Upon cardiac injury, activated cardiac fibroblasts infiltrate the injury site, transforming into myofibroblast cells that proliferate and deposit collagen and other extracellular matrix (ECM) components, ultimately leading to fibrosis [26]. Our present study contributes valuable insights into the positive effects of neferine in attenuating Ang II-induced AF and fibrosis. We observed that Ang II treatment significantly activated the TGF-β/p-Smad2/3 signaling pathway, resulting in atrial fibrosis marked by increased mRNA expression of collagen I and III. These effects were notably inhibited by neferine. Importantly, our data conclusively demonstrate that neferine's attenuation of Ang II-induced AF, atrial dilation, fibrosis, and oxidative stress is dependent on HO-1.

Our study possesses certain limitations since the Ang II-induced mouse model of AF may not entirely replicate the complexity of the clinical environment. AF is a multifaceted disease with numerous predisposing factors capable of triggering its occurrence. In addition to elevated Ang II concentration and blood pressure, risk factors such as genetic mutations, obesity, hyperlipidemia, sleep apnea, smoking, alcohol consumption, drug administration, and a lack of physical activity are all associated with the development of AF [27]. Whether the conclusions drawn from our neferine study can be extrapolated to AF caused by other reasons remains a subject for further investigation. Nevertheless, the outcomes of our study affirm the protective role of neferine in Ang II-induced AF, with the activation of the nrf2/HO-1 pathway identified as the underlying molecular mechanism. The inhibitory effects of neferine on oxidative stress and fibrosis were nullified when HO-1 was inhibited by ZnPP, highlighting HO-1 as a potential therapeutic target for AF.
